# Annual Cambial Rhythm in *Pinus halepensis* and *Pinus sylvestris* as Indicator for Climate Adaptation

**DOI:** 10.3389/fpls.2016.01923

**Published:** 2016-12-26

**Authors:** Peter Prislan, Jožica Gričar, Martin de Luis, Klemen Novak, Edurne Martinez del Castillo, Uwe Schmitt, Gerald Koch, Jasna Štrus, Polona Mrak, Magda T. Žnidarič, Katarina. Čufar

**Affiliations:** ^1^Slovenian Forestry Institute, University of LjubljanaLjubljana, Slovenia; ^2^Department of Geography and Regional Planning, University of ZaragozaZaragoza, Spain; ^3^Department of Ecology, University of AlicanteAlicante, Spain; ^4^Johann Heinrich von Thünen Institute – Thünen Institute of Wood ResearchHamburg, Germany; ^5^Department of Biology, Biotechnical Faculty, University of LjubljanaLjubljana, Slovenia; ^6^Department of Biotechnology and Systems Biology, National Institute of Biology, University of LjubljanaLjubljana, Slovenia; ^7^Department of Wood Science and Technology, Biotechnical Faculty, University of LjubljanaLjubljana, Slovenia

**Keywords:** Aleppo pine, cambium, light microscopy, Mediterranean environment, Scots pine, temperate environment, transmission electron microscopy, xylem

## Abstract

To understand better the adaptation strategies of intra-annual radial growth in *Pinus halepensis* and *Pinus sylvestris* to local environmental conditions, we examined the seasonal rhythm of cambial activity and cell differentiation at tissue and cellular levels. Two contrasting sites differing in temperature and amount of precipitation were selected for each species, one typical for their growth and the other represented border climatic conditions, where the two species coexisted. Mature *P. halepensis* trees from Mediterranean (Spain) and sub-Mediterranean (Slovenia) sites, and *P. sylvestris* from sub-Mediterranean (Slovenia) and temperate (Slovenia) sites were selected. Repeated sampling was performed throughout the year and samples were prepared for examination with light and transmission electron microscopes. We hypothesized that cambial rhythm in trees growing at the sub-Mediterranean site where the two species co-exist will be similar as at typical sites for their growth. Cambium in *P. halepensis* at the Mediterranean site was active throughout the year and was never truly dormant, whereas at the sub-Mediterranean site it appeared to be dormant during the winter months. In contrast, cambium in *P. sylvestris* was clearly dormant at both sub-Mediterranean and temperate sites, although the dormant period seemed to be significantly longer at the temperate site. Thus, the hypothesis was only partly confirmed. Different cambial and cell differentiation rhythms of the two species at the site where both species co-exist and typical sites for their growth indicate their high but different adaptation strategies in terms of adjustment of radial growth to environmental heterogeneity, crucial for long-term tree performance and survival.

## Introduction

Radial growth of woody plants is a result of the activity of secondary meristems. It is controlled by a complex of endogenous and exogenous factors, such as temperature, precipitation, light intensity and duration (Waisel and Fahn, 1965; Creber and Chaloner, 1984). There are many morphological and physiological strategies of woody species to renew their vascular tissues and survive in different environments (Lachenbruch and McCulloh, 2014). Information on xylem and phloem cell characteristics is necessary (Gričar et al., 2015) to increase current knowledge about the plasticity of secondary growth that allows trees to adapt to specific environmental regimes (e.g., drought or low temperatures) (Rowe and Speck, 2005; Agusti and Greb, 2012).

Formation of xylem and phloem tissues is initiated in the vascular cambium, composed of meristematic fusiform and ray cells (Larson, 1994). In temperate zones with cold winters and warm summers, the vascular cambium of trees undergoes periodic annual cycles of activity and dormancy (Lachaud et al., 1999). Winter dormancy (the period with no cell division) is an important adaptive mechanism for the survival of perennial plants in temperate and cold climates. Cambial cell division generally starts in spring and continues until late summer or early autumn (Larson, 1994; Rossi et al., 2014). The periodicity of cambial activity is characterized by clear annual growth ring boundaries due to changes in the morphology of earlywood and latewood cells, and by winter dormancy. The quality and quantity of the wood thus reflect the seasonal dynamics of wood formation processes in trees (Begum et al., 2013; Schmitt et al., 2016).

The typical Mediterranean climate is characterized by hot and dry summers and mild winters, with a pronounced rainfall maximum in winter or autumn. Since cambium does not exhibit a regular dormancy period and shows a great variability in meristematic activity, annual tree rings are not always formed, which is reflected in the specific xylem structure in trees from this region, as for example intra-annual density fluctuations, missing and dark rings (e.g., Cherubini et al., 2003; De Luis et al., 2011b; Novak et al., 2016b; Zalloni et al., 2016). During the period of low cambial activity, cell production might still occur but at a very low rate (De Luis et al., 2007, 2011a) and it can only be checked with proper fixation of tissues and their examination at high resolution by transmission electron microscopy (e.g., Prislan et al., 2011). Cambial cell production on xylem and phloem sides in trees from Mediterranean regions is temporarily not synchronous, as is typical of temperate trees. The radial growth in trees from mild climates often does not exhibit a distinct annual cycle, suggesting that variation in cambial seasonality and cell differentiation is a species and site-specific phenomenon (Barnett, 1971; Gričar et al., 2016).

In addition to climatic conditions, the annual rhythm of cambial activity among other things depends on tree vigor, the part of a tree and tree age (Larson, 1994). Moreover, in tree species with a wide distribution range, differences in cambial activity can also result from genetic diversity (Liphschitz and Lev-Yadun, 1986; Nardini et al., 2014; Vieira et al., 2015). In well-adapted woody plants composing the climatic climax community in a given area, the annual rhythm of cambial activity usually parallels or overlaps that of the climatic rhythm. In evergreens, the cambial rhythm is preserved even when plants grow under climatic conditions, which differ from those of their natural habitat, indicating that the control of cambial activity in these species is primarily endogenous (Waisel and Fahn, 1965; Camarero et al., 2010). However, this holds true only in mild winter climatic conditions that are favorable for cambial activity, while in the case of harsh climatic conditions compared to those of the tree’s native environment, climatic factors may override genetic ones (Barnett, 1971). Comparison of the patterns of the annual rhythm of cambial activity and cell differentiation, which is reflected in the structure of xylem and phloem increments, can thus show the degree of adaptation of tree species to local environmental conditions (Gričar et al., 2016).

*Pinus sylvestris* is a temperate conifer with an extremely wide distribution, occupying a broad range of habitats across the entire Eurasian continental landmass, whereas *Pinus halepensis* forests cover extensive areas in the Mediterranean area (Fady et al., 2003; Mátyás et al., 2004). These two *Pinus* species co-exist in some marginal parts of their distributional range. Because of their ecological and economic importance, the cambial activity of both species has been monitored in different locations in Europe (e.g., De Luis et al., 2007; Camarero et al., 2010; Oberhuber and Gruber, 2010; Seo et al., 2011). Potentially, the bimodal radial growth pattern of *P. halepensis* (e.g., De Luis et al., 2007, 2011a; Camarero et al., 2010; Novak et al., 2016a) is generally distinct from the unimodal pattern of *P. sylvestris* (Rossi et al., 2008; Seo et al., 2008; Camarero et al., 2010; Martinez Del Castillo et al., 2016). Despite numerous studies on seasonal patterns of cambial activity in these two species, comparisons of the radial growth in coexisting species are not so common, particularly when inspected at tissue and ultrastructural levels. Detailed studies of ultrastructural and biochemical changes in cambium in relation to alternate periods of meristematic activity have been predominantly carried out in species from the temperate zone (Lachaud et al., 1999). Little is therefore known about the intraspecific plasticity of cambial phenology from contrasting locations in these two species, despite its relevance to tree growth and survival in different ecosystems.

In order better to understand the adaptation of *P. halepensis* and *P. sylvestris* to local environmental conditions, we examined their intra-annual rhythm of cambial activity or inactivity. On samples properly prepared for light and transmission electron microscopy, we observed seasonal changes in the cambial cells at tissue and cellular levels. Mature *P. halepensis* trees from Mediterranean and sub-Mediterranean sites, and *P. sylvestris* from sub-Mediterranean and temperate sites with different climatic regimes were included in the study. One of the selected sites (i.e., Mediterranean for *P. halepensis* and temperate for *P. sylvestris*) was typical for each species and the other (sub-Mediterranean) represented border climatic conditions in which the two species coexist. We hypothesized that, irrespective of the local climatic conditions, cambial rhythms and cell differentiation patterns in the two species at the latter, sub-Mediterranean site with mild winters would be similar as in trees at typical sites for their growth.

## Materials and Methods

### Study Site Characteristics

**FIGURE 1:**
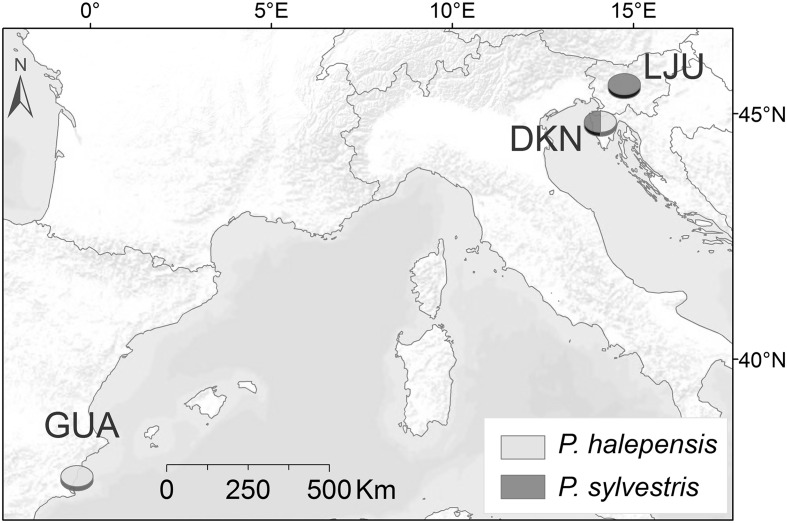
**Map with sampling locations: Guardamar del Segura (GUA), Dekani (DKN), and Ljubljana (LJU)**.

The study was conducted at three forest sites differing in climatic conditions. One site was selected in Spain and two in Slovenia (**Table 1**; **Figure 1**).

**Table 1:** Description of the selected forest sites.

Site	Geographic coordinates	Altitude (m a.s.l.)	Mean annual temperature (C)	Annual sum of precipitation (mm)
Guardamar del Segura (GUA)	38°6′ N, 0°40′ W	5	18.1	305
Dekani (DKN)	45°32′ N, 13°49′ E	90	12.9	984
Ljubljana (LJU)	46°03′ N, 14°28′ E	323	10.2	1370

*Weather data present the long-term average for the period 1950 to 2011*.

The Spanish site, Guardamar del Segura (GUA), is a sand dune ecosystem located on the coast with semi-arid Mediterranean climate, with mean annual temperature of 18.1°C and annual precipitation of 305 mm for the period 1950–2011. The actual forest was planted 100 years ago to stabilize the dunes and prevent dune expansion. It is a mixed plantation of *P. halepensis* Mill. and *Pinus pinea* L. including also some areca palms and eucalyptus trees planted near the coast line.

In Slovenia, one site was selected in the Slovene littoral, the northern part of the Istrian peninsula, with sub-Mediterranean climate, characterized by 12.9°C mean annual temperature and 984 mm mean annual sum of precipitation (meteorological station Portorož for the period 1950–2011). The forest site is located near Dekani (DKN) (few kilometers from the port of Koper) and belongs to the *Orno-Cotino-quercetum pubescentis* forest association with *Quercus pubescens* Willd. as the potentially predominant natural species. *P. halepensis* grows on a sun exposed flysch slope with shallow rendzina soil, where other *Pinus* species, such as *Pinus nigra* J. F. Arnold and *P. sylvestris* L., also grow (Dakskobler et al., 2012).

The third, temperate site was selected in the central part of Slovenia on Rožnik hill in the city of Ljubljana (LJU). It is located in a privately owned forest devoted to its natural development. The forest site belongs to the *Blechno Fagetum* forest association, with *Fagus sylvatica* L. and *Quercus petraea* (Matt.) Lieb. as predominant tree species. The climate is humid continental. According to the climate record from the nearby meteorological station of Ljubljana for the period 1950–2011, the mean annual temperature is 10.2°C and average annual sum of precipitation of 1370 mm.

### Sample Collection and Preparation

*Pinus halepensis* trees were chosen at the typical Mediterranean site GUA. Coexisting *P. halepensis* and *P. sylvestris* trees were selected at the sub-Mediterranean site DKN, while at the temperate site LJU, only *P. sylvestris* trees were sampled. At each site, two dominant or co-dominant healthy trees aged around 70 years were selected per species. Larger blocks of intact tissues were collected with chisel and knife (Gričar, 2007) for cellular and ultrastructural observations by light microscope (LM) and transmission electron microscopy (TEM). One sample per tree was collected between December 2009 and January 2011 and in total 38 samples were collected. Exact sampling dates for each site are given in **Table 2**. All samples were collected from tree stems at approximately 1.3 m above ground. They were taken at least 10 cm apart from each other to avoid wound effects. Each intact tissue sample contained part of phloem tissue, cambium and a minimum of two of the last-formed xylem rings.

**Table 2:** Sampling dates and day of the year (DOY) and sampling locations (GUA, Guardamar del Segura; DKN, Dekani and LJU, Ljubljana).

Sampling	DOY/Date					
	GUA		DKN		LJU	
1	348	14 December 2009	349	15 December 2009		
2	45	14 February 2010	46	15 February 2010		
3	80	21 March 2010	81	22 March 2010	81	22 March 2010
4	129	9 May 2010	130	10 May 2010	130	10 May 2010
5	185	4 July 2010	186	5 July 2010	186	5 July 2010
6	276	3 October 2010	277	4 October 2010	277	4 October 2010
7	23	23 January 2011	24	24 January 2011	24	24 January 2011

After removal from the stem, the samples were reduced with sharp razor blades to less than 2 mm in thickness in order to ensure proper fixation (Bozzola and Russell, 1999). The reduced samples contained cambium and the youngest xylem and phloem portions. To observe the ultrastructural seasonal changes in the cambial cells, the samples were first fixed for 1 day in 5% glutaraldehyde, 8% paraformaldehyde, and 0.3 M cacodylate buffer (pH 7.3). They were washed in three subsequent washes with 0.1 M cacodylate buffer for 25–30 min each and post-fixed for one additional day in a 2% aqueous osmium tetroxide solution. Thereafter, the samples were washed again three times (for 25–30 min) in 0.1 M cacodylate buffer (pH 7.3) and dehydrated through a graded series of acetone (from 30 to 100% increasing by 10%; for 15 min in each concentration). After dehydration, the cambium samples were infiltrated with a (50:50) mixture of acetone and Spurr’s (1969) epoxy resin for at least 2 h and then for 24 h in 100% epoxy resin. Samples were then embedded in epoxy resin using silicone molds and polymerised at 65°C for 3 days.

Semi-thin (0.5–1 μm thick) and ultra-thin (70–90 nm thick) transverse sections of the cambial region were cut with a Reichert Ultracut S ultramicrotome (Leica) using a glass or diamond knife, respectively. Semi-thin sections were stained with toluidine blue (Merck, Darmstadt, Germany) (0.5%) or Richardson’s stain (1:1 mixture of 1% Azure II and a solution of 1% sodium tetraborate and 1% Methylene Blue) and examined with a Nikon Eclipse E800 LM equipped with a DS-Fi1 digital camera. Histometrical analyses were performed using a NIS Elements BR3 image analysis system (Tokyo, Japan) with an accuracy of 1.14 and 0.46 μm/px at 4 and 10 times magnification, respectively. Ultra-thin sections were placed on copper grids and post-stained with 1% uranyl acetate and 10% lead citrate and examined with a Philips CM 100 TEM at an accelerating voltage of 80 kV (e.g., Farrar and Evert, 1997; Frankenstein et al., 2005; Prislan et al., 2011). Images were captured with BioScan 792 camera using Digital Micrograph software.

### Tissue Analyses and Measurements

On the semi-thin sections, the number of cell layers in the cambium, as well as the number of developing xylem cells in the stage of expansion, and secondary cell-wall formation along the same radial file was assessed by LM. Cambial cells were distinguished from xylem and phloem cells based on their smaller radial diameter. According to Plomion et al. (2001), cambium activity was identified and interpreted within the context of the multi-seriate concept, meaning that the cambium comprises both the cambial initials and the xylem and phloem mother cells.

Seasonal changes in the cytoplasm of cambial cells (Farrar and Evert, 1997; Rensing and Samuels, 2004; Prislan et al., 2011) and changes in the architecture of cambial cell walls (Chen et al., 2010) were examined by TEM. In order to identify different stages of cambial dormancy, activity and productivity, terminology as suggested by Prislan et al. (2013) was used.

### Weather Conditions at Sampling Time

Mean, maximum and minimum daily air temperature for the 10-day period before the sampling date were averaged in order to compare the weather conditions at the sampling sites. The length of the period was based on observations by Gričar et al. (2006), who revealed by heat treatment experiments that a 10-day period of increasing temperature seems to determine cambial reactivation after winter dormancy. In Slovenia, daily weather data were obtained from the Environmental Agency of the Republic of Slovenia from weather stations Ljubljana Bežigrad (46°03′ N, 14°30′ E, 299 m a.s.l.) for the temperate site (LJU) and Portorož (45°28′ N, 13°36′ E, 2 m a.s.l.) for the sub-Mediterranean site (DKN). For the Mediterranean site GUA, weather data were obtained from the Spanish Meteorological Agency (AEMET) from Guardamar del Segura station (38°5′ N, 0°39′ W, 27 m a.s.l).

During winter sampling in December 2009, February 2010, March 2010 and January 2011, the lowest average temperature was recorded at LJU (-1 to 7°C), followed by DKN (4–7°C) and the highest temperature was recorded at GUA (around 12°C). In May, July and October 2010, the average temperature before sampling was similar (14–23°C) at LJU and DKN, and slightly higher at GUA (18–24°C). The lowest amount of precipitation in the period of 1 month before each sampling date was detected at GUA followed by DKN and the highest at the temperate site LJU (**Table 3**). Annual amount of precipitation in 2009 and 2010 was higher than the long-term average at most sites. At GUA annual amount of precipitation was 37% (419 mm) and 15% (352 mm) higher in 2009 and 2010 respectively, at DKN annual precipitation in 2009 were 5% (932 mm) lower, however in the subsequent year 41% (1394 mm) higher than the long-term average. At LJU annual precipitation in 2009 and 2010 were 2% (1405 mm) and 31% (1798 mm) higher than long-term average.

**Table 3:** Average mean, maximum and minimum daily temperature of 10-day period before sampling and sum of precipitation for the period 1 month before sampling at the Mediterranean (GUA), sub-Mediterranean (DKN) and temperate (LJU) sites.

Sampling	DKN				LJU				GUA			
	avr.T	max.T	min.T	PP	avr.T	max.T	min.T	PP	avr.T	max.T	min.T	PP
1	7.3	12.7	3.5	131.3	4.4	6.8	2.4	111.5	12.6	17.9	7.2	52.4
2	4.1	8.1	1.0	51.8	-0.8	0.9	-2.1	64.3	11.7	15.3	8.1	20
3	7.0	12.7	2.4	35.9	6.8	12.2	1.3	58	11.1	15.4	6.7	39.7
4	15.9	20.3	12.3	61	14.2	18.3	10.9	78.2	17.7	22.4	13.0	23.4
5	23.4	29.3	17.0	66.8	22.7	29.4	16.2	113.1	23.9	29.2	18.5	67.8
6	15.4	21.0	12.1	257.8	13.7	16.7	11.4	425.4	22.1	27.6	16.6	10.8
7	3.8	6.4	1.7	39	1.2	4.9	-1.5	111.6	10.1	14.2	5.9	2.8

## Results

### Cambial Rhythm of *P. halepensis* at the Mediterranean Site (GUA)

**FIGURE 2:**
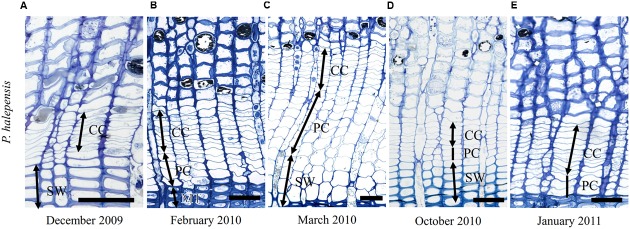
**Light micrographs of the cambial region in *Pinus halepensis* from Mediterranean site GUA on sampling dates between December 2009 and January 2011 (A–E)**. The number of cambial cells (CC) varied among the sampling dates. Below the cambium, xylem cells in different stages of differentiation can be observed; i.e., enlarging cells (PC), cells in the stage of secondary wall formation (SW) and fully developed mature cells (MT). Scale bars 50 μm.

**FIGURE 3:**
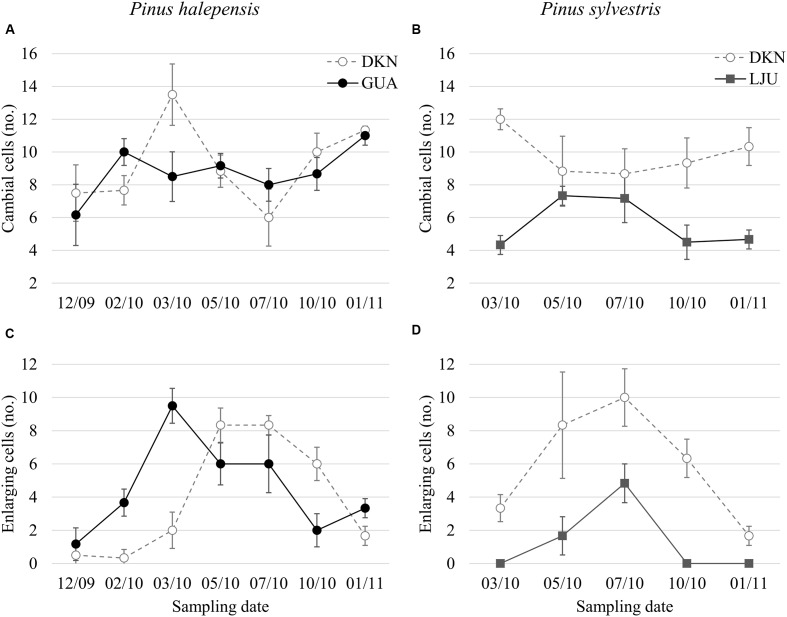
**Average number of cambial cells** **(A,C)** and enlarging tracheids **(B,D)** in *P. halepensis* and *Pinus sylvestris* at the sampling dates as observed with LM. Bars represent standard deviations. Study sites are denoted by filled circles for the Mediterranean (GUA), open circles for the sub-Mediterranean (DKN) and filled rectangles for the temperate site (LJU).

At Guardamar (GUA) in Spain, the first sampling was performed around the winter solstice in 2009. At that time, the cambium was on average six cell layers wide. Adjacent to the cambium, one or two layers of enlarging xylem cells, as well as cells in various stages of secondary wall formation, were observed (**Figure 2A**). Based on their radial dimensions and wall thicknesses, they were classified as differentiating latewood cells. In the middle of February 2010, the number of cells in the cambium increased to up to 10 layers (**Figure 3A**). Two to three enlarging xylem cells had significantly larger radial dimensions than cambium cells and therefore resembled differentiating earlywood cells (**Figure 2B**). One month later, the forming xylem ring of 2010 was on average 14 cells wide, with the initial tracheids being already in the stage of secondary wall formation (**Figure 2C**). On the subsequent sampling dates in May, July, October 2010 and January 2011, the number of cambial cells varied between 8 and 11 layers (**Figures 2D,E**). However, the number of enlarging tracheids changed significantly; in May and July six layers were present on average and in October and January 2–3 layers (**Figure 3C**), indicating differences in the rate of cell production between warmer and colder periods of the year.

**FIGURE 4:**
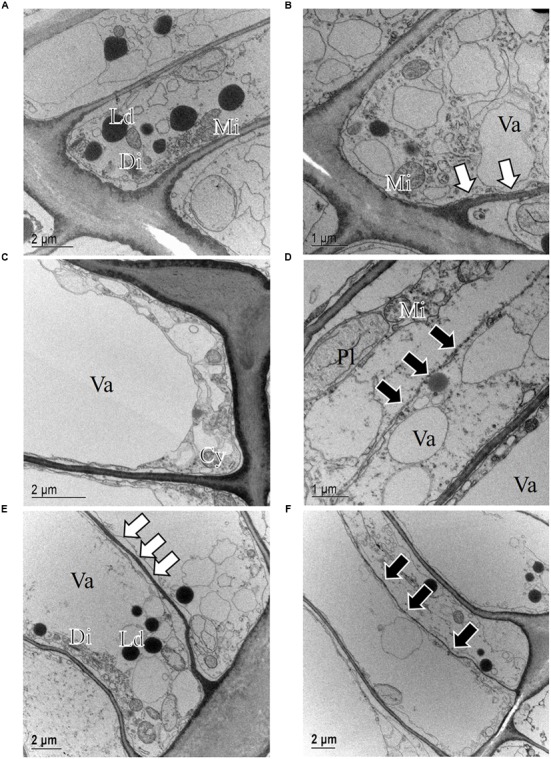
**Transmission electron micrographs of fusiform cambial cells (CC) in *P. halepensis* trees from Mediterranean site GUA in December 2009 (A,B)**, May 2010 **(C)**, October 2010 **(D)**, and January 2011 **(E,F)**. In December 2009 **(A,B)**, the ultrastructure of fusiform cambium cells was different from that from other sampling dates; cytoplasm appeared denser, containing numerous lipid droplets (Ld), Golgi stacks (Di), plastids (Pl) and mitochondria (Mi); in some cells, newly formed cell walls (white arrows) were observed. In May 2010 **(C)** and October 2010 **(D)** as well as in January 2011 **(E,F)**, cambium cells in *P. halepensis* from the Mediterranean site were active and contained large vacuoles (Va), occupying most of the lumen, and a thin parietal layer of cytoplasm (Cy). At these dates, cell plates (white arrows) and new cell walls (black arrows) were observed.

Light microscope observations revealed that the cambium of *P. halepensis* at GUA was productive in most of the samples collected throughout the year (**Figures 2B–E**). TEM revealed that fusiform cambial cells were characterized by large central vacuoles, whereas other cell organelles were aggregated in narrow cytoplasmic strands close to the cell wall (**Figure 4C**). The cell walls were thin and newly formed cell plates were visible in some cells (**Figures 4D,F**). In actively dividing cambial cells, Golgi stacks were surrounded by numerous secretory vesicles and mitochondria were numerous with mostly spherical shape. We noticed that lipid droplets were less frequent in cambial cells from samples collected in spring and summer months than those in winter months (**Figures 4C–F**). Although plastids were present in fusiform cambial cells, they were mainly located in ray cambial cells and often contained starch grains.

The only sampling date at this location was in mid-December 2009, when the cambium was expected to be in a dormant or non-productive stage. TEM observations of fusiform cambial cells from that sampling date confirmed distinct differences in the ultrastructure compared to the other samples (**Figures 4A,B**). Some fusiform cambial cells on that sampling date had a dense cytoplasm and small vacuoles, an increased number of lipid droplets and relatively thick cell walls. However, vacuoles in other fusiform cambial cells occupied most of the lumen, which is a characteristic of active cambial cells. In addition, thinner tangential cell walls, indicating recent division, were also observed in some cells (**Figure 4B**).

### Cambial Rhythm of *P. halepensis* and *P. sylvestris* at the sub-Mediterranean Site (DKN)

**FIGURE 5:**
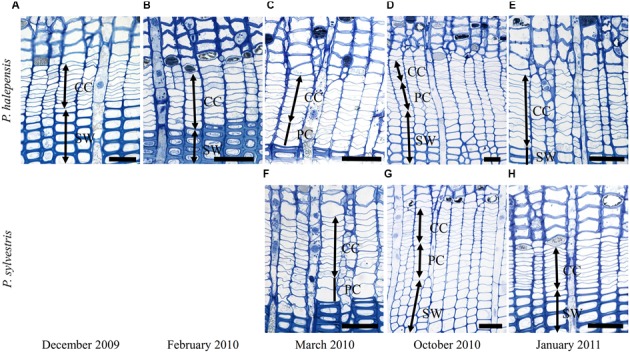
**Light micrographs of the cambial region in *P. halepensis* (A–E)** and *P. sylvestris* **(F–H)** from sub-Mediterranean site DKN between December 2009 and January 2011. The number of cambial cells (CC) in *P. halepensis* is similar in December 2009 **(A)** and February 2010 **(B)**. At that time, tracheids of the previous growth ring were still in the stage of secondary wall formation (SW). In March **(C,F)** cambium was productive in both species, as indicated by an increased number of enlarging cells (PC). In October 2010 **(D,G)** cambium was still productive in both species and xylem cells in all stages of differentiation can be observed. In January 2011 **(E,H)**, cambium is apparently not productive, although xylem cells are still in the SW formation stage. Scale bars 50 μm.

**FIGURE 6:**
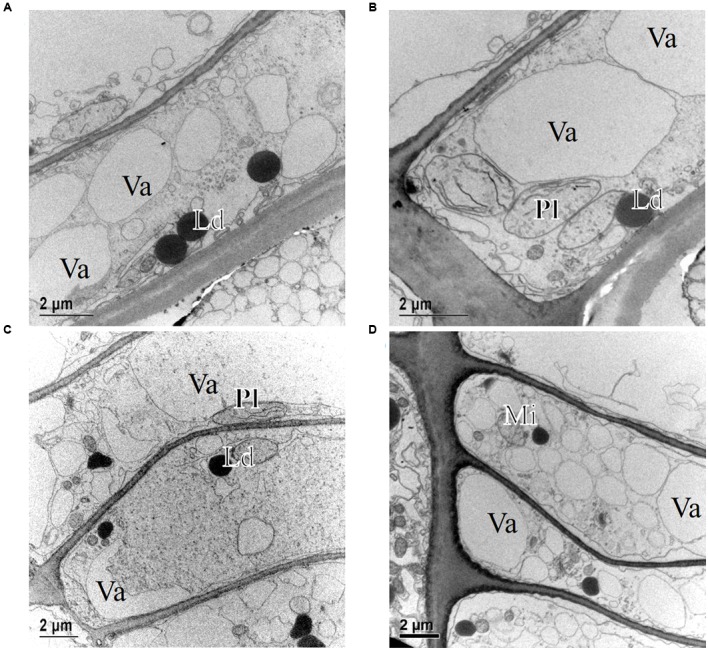
**Transmission electron micrographs of fusiform cambial cells (CC) in *P. halepensis* and *P. sylvestris* trees from the sub-Mediterranean site (DKN)**. In December 2009 **(A,B)** and January 2011 **(C)**, fusiform cambium cells in *P. halepensis* were dormant, as characterized by thick cell walls and dense cytoplasm with numerous small vacuoles (Va), large and numerous lipid droplets (Ld), plastids (Pl) without starch grains and round mitochondria (Mi). A similar situation was also observed in fusiform cambial cells in *P. sylvestris* in January 2011 **(D)**.

At the sub-Mediterranean site Dekani (DKN) in Slovenia, both *P. halepensis* and *P. sylvestris* were sampled concurrently. The number of cambial cells in *P. halepensis* in December 2009 was similar to that at the GUA site (six cell layers) (**Figure 3A**). However, at the sub-Mediterranean site, the number of cambial cells remained almost unchanged also in February 2010. At both sampling dates, one to two enlarging earlywood tracheids adjacent to the cambium were observed in some radial files, which were characterized by larger radial dimensions (**Figures 5A,B**). The highest number of cambial cells was observed in March (on average 13 layers) (**Figure 5C**) and the lowest in July 2010 (six cells on average) (**Figure 3A**). In October 2010 and January 2011, the number of cambium cells increased again to 10 and 11 layers, respectively (**Figures 3A** and **5D,E**). The number of enlarging cells followed a bell-shaped curve; the number increased from March until May and then decreased from July until October 2010 (**Figure 3C**). During the productive period (between March and October), on average three more enlarging cells in a radial file were observed at DKN than at GUA (**Figure 3C**). In the samples collected in March, May, July, and October 2010, the cambium of *P. halepensis* at DKN was highly productive, as indicated by organelle appearance and a high number of enlarging tracheids close to the cambium. The cambium in December 2009 and January 2011 appeared to be dormant; the cells had thick walls and dense cytoplasm, with numerous small vacuoles (**Figures 6A–C**). Furthermore, the number of lipid droplets was higher than in March and October samples, whereas starch grains in plastids could not be seen in either fusiform or ray cambial cells.

The first samples of *P. sylvestris* at DKN were collected in mid-March 2010. At that time, on average 12 layers of cambial cells and two layers of enlarging cells were already observed (**Figures 3B,D**). Due to proper fixation, newly formed cell walls (and cell plates) were observed in fusiform and ray cambial cells, even with LM (**Figure 5F**). During the summer months, as well as in October, the cambium was active, as indicated by a high number of cambial cells (on average nine layers) and the presence of enlarging tracheids (**Figure 5G**). In January 2011, the cambium appeared dormant but still contained on average 10 cell layers. On the xylem side, undifferentiated latewood cells, as well as cells resembling enlarging stages adjacent to the cambium, with larger radial dimensions and thin cell walls were observed (**Figure 5H**). However, neither new cell plates nor recently formed (thinner) cell walls were observed with TEM. In addition, the appearance and distribution of cell organelles were typically dormant (**Figure 6D**), although some cells already contained large central vacuoles, indicating the beginning of transition from dormancy to activity.

### Cambial Rhythm of *P. sylvestris* at the Temperate Site (LJU)

**FIGURE 7:**
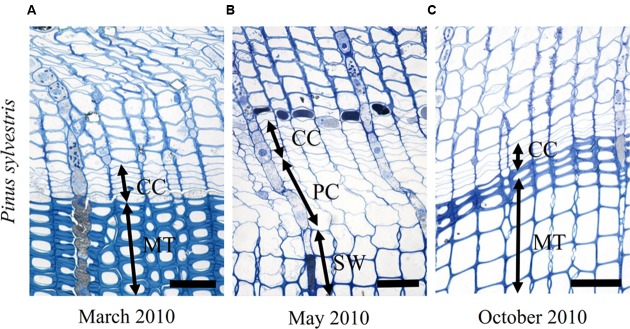
**Light micrographs of the cambial region in *P. sylvestris* from the temperate site (LJU) in March 2010 (A)**, May 2010 **(B)**, and October 2010 **(C)**. In March **(A)** four to five cell layers can be observed in the cambium and below the cambium mature (MT) latewood tracheids. In May **(B)**, the cambium was productive, as indicated by xylem cells in several stages of differentiation; enlargement (PC) and secondary wall formation (SW). In October **(C)** cambial production had already ceased and mature latewood cells were observed below the cambium. Scale bars 50 μm.

**FIGURE 8:**
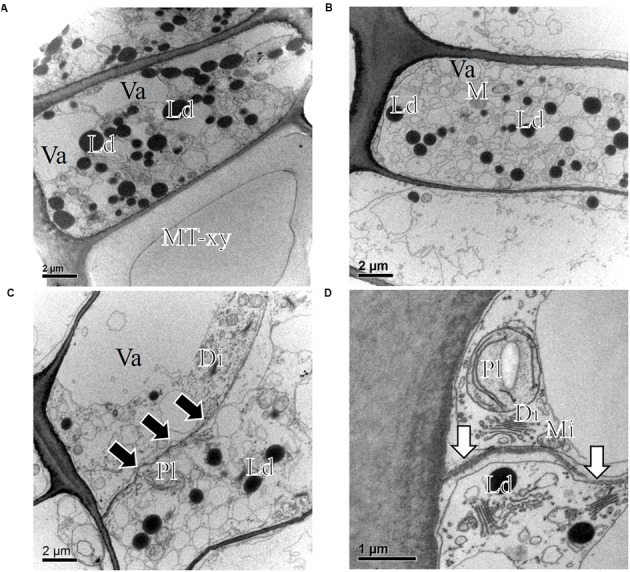
**Transmission electron micrographs of fusiform cambial cells (CC) in *P. sylvestris* trees from the temperate site (LJU)**. In March 2010 **(A)** and January 2011 **(B)**, fusiform cambium cells in *P. sylvestris* were dormant, as characterized by thicker cell walls, dense cytoplasm with several small vacuoles (Va) and numerous small lipid droplets (Ld). Mature xylem cells (MT-xy) were present below the cambium in the samples collected in March 2010. In May **(C)** and July **(D)** the cambium was active; dividing cells with new cell plates (black arrows) were visible **(C)** and cell wall formation (white arrows) **(D)**, plastids (Pl) containing starch grains and Golgi stacks (Di) were numerous and located mostly near newly formed cell walls.

At the temperate site Ljubljana (LJU) in Slovenia, in March 2010 and January 2011 the cambial cells in *P. sylvestris* were typically dormant, consisting of around four cell layers (**Figures 3B** and **7A**). When comparing the ultrastructure of dormant cambium in *P. sylvestris* from the sub-Mediterranean (DKN) with the temperate (LJU) site, the major difference was in the number and size of lipid droplets; they were more numerous and larger at LJU (**Figures 8A,B**). The higher number of cambial cells in May and July 2010, presence of enlarging cells (**Figure 7B**) and also the ultrastructure of cambium cells (**Figures 8C,D**) indicate that the cambium was active at that time. In October 2010, the number of cambial cells decreased to on average four layers and adjacent xylem cells were fully differentiated (**Figure 7C**). TEM showed that the walls of cambial cells were still relatively thin and lipid droplets were less numerous than in March 2010 or July 2011, suggesting that the cambium was in transition from active to dormant at that time.

## Discussion

### Cambial Rhythm of *P. halepensis* and *P. sylvestris* in Different Environments

The combination of TEM and LM observations enabled investigation of cambial activity on the ultrastructural level (organelle distribution in its dormant and active stages) and cambium productivity at the tissue level (i.e., production of new xylem and phloem cells) (Prislan et al., 2013). These two stages of the cambium are not synchronous; activity may start up to 1 month before cambial productivity (Prislan et al., 2011).

**FIGURE 9:**
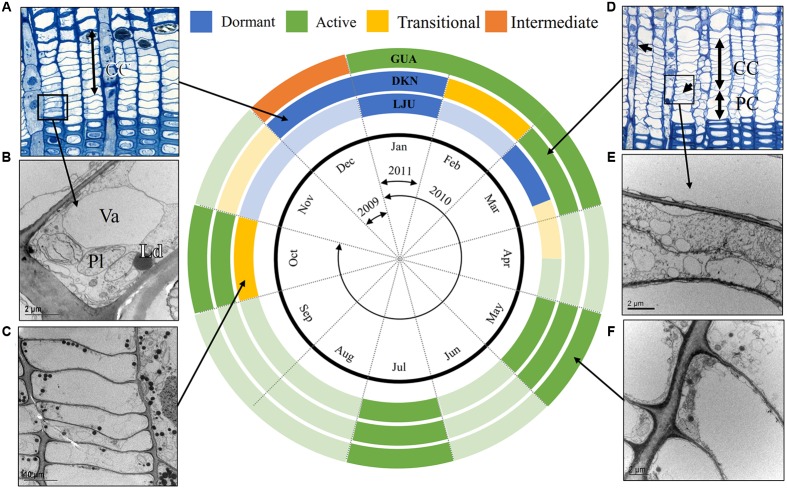
**Schematic representation of the cambial rhythm in *P. halepensis* and *P. sylvestris***. Three circles represent the selected sites; Mediterranean (GUA), sub-Mediterranean (DKN) and temperate (LJU). The black circle represents a year divided into 12 months and the arrows indicate the sampling period between December 2009 and January 2011. Active, transitional, dormant and “intermediate” cambium is marked as green, yellow, blue, and orange, respectively. In months in which no sampling was performed, a hypothetical cambium stage is estimated based on our previous studies (e.g., De Luis et al., 2007; Gričar et al., 2014) and thus indicated by a lighter color. In the dormant stage, cambium is characterized by cells with small radial dimensions and relatively thick cell walls, as seen under LM **(A)**. As observed with TEM, dormant cambium cells contain a dense cytoplasm with numerous small vacuoles **(B)**. During activity-dormancy transition, the large central vacuole fragment into a number of smaller ones and the number of lipid droplets increases **(C)**. Cell production can be observed by an increased number of cambium cells (CC) and the presence of enlarging cells (PC) **(D)**. Newly formed cell plates can be observed with TEM **(E)**. Active fusiform cambial cells have a large central vacuole and a thin layer of cytoplasm confined to the cell wall **(F)**.

At the Mediterranean site GUA in Spain, the cambium of *P. halepensis* was never clearly dormant (**Figure 9**); only in samples collected around the winter solstice (December 2009) did the cambium seem to be in an “intermediate” stage, since some cells appeared to be in a dormant and others in an active stage. The latter cells could presumably quickly return to cell division in the case of favorable climatic conditions. In samples collected in winter (January 2011), the cambium cells were again active. Since the average temperature 10 days before these two sampling dates was 12 and 10°C, respectively, it is suggested that temperature is not a limiting factor for cambium productivity at the selected site. The finding that the cambium may not be dormant in the case of mild winter conditions is not new. It was already reported over 40 years ago for young *Pinus radiata* from North Island of New Zealand (Barnett, 1971). Although no new xylem cells were produced for a short period during the winter, cambial cells did not seem to be dormant and might have continued cell production on the phloem side (Barnett, 1971; Gričar et al., 2016). It is assumed that in coastal Mediterranean areas with mild winters and dry summers, cambial activity might be continuous, with no dormancy in the cold season (Cherubini et al., 2003; De Micco et al., 2016).

At the sub-Mediterranean site DKN, however, cambium seasonality in *P. halepensis* appeared to be similar to that in co-existing *P. sylvestris* trees, in which cells were dormant during the winter (**Figure 9**). The average temperature in December 2009, February 2010, and January 2011 at this site was lower than 8.4°C, which, according to Rossi et al. (2008), is the mean threshold temperature for the onset of cambial xylem cell production in the case of temperate and boreal conifer species. In (sub)-Mediterranean species, reduced cambial activity is related to low temperature in the autumn-winter period or drought during summer months (Lev-Yadun, 2000; Cherubini et al., 2003; Camarero et al., 2010). In summer 2010, a reduced rate of cell division, resulting in a reduced number of cambial and expanding cells due to drought, were not evident at the (sub)-Mediterranean sites GUA and DKN. This can be explained by the above average amount of precipitation at both Mediterranean sites in 2010, so it still remains open whether the pattern would remain the same in the case of a dry year. Our results are therefore in line with previous reports on the plastic cambial behavior of *P. halepensis*, which is closely linked to current environmental conditions, such as low temperature or water deficit (Liphschitz and Lev-Yadun, 1986; Camarero et al., 2010). This species is also known to adapt to water shortage by reducing water use during drought by stomatal closure (Borghetti et al., 1998). The year-to-year variability of the Mediterranean climate, coupled with high intra-specific plasticity, may thus explain the diverse phenological growth patterns of *P. halepensis* (Castro-Díez and Montserrat-Martí, 1998).

High variability in cambial phenology can also be observed in *P. sylvestris*. According to Seo et al. (2011), who investigated cambial productivity in *P. sylvestris* at two sites in Northern Finland, cambium was productive for 9 weeks in late spring and early summer at the southern site near the Arctic Circle and only 7 weeks at the northern site near the Arctic tree line. In contrast, the cambial productivity of *P. sylvestris* proved to last 11–13 weeks at xeric and dry-mesic sites in the Austrian Alps (Gruber et al., 2010; Oberhuber and Gruber, 2010). We found that the cambial phenology of *P. sylvestris* at the temperate site LJU differed significantly from that at the sub-Mediterranean site DKN (**Figure 9**). Although the average temperature of the 10-day period before sampling was similar (around 7°C) at the two sites in March 2010, cambial productivity had already occurred at the sub-Mediterranean site, whereas the cambium was still dormant at the temperate site. A similar situation was observed in October 2010, when the cambium was still productive at the sub-Mediterranean site, while the cambium was in the stage of transition from activity to dormancy at the temperate site. This indicates that cell production ceased few weeks earlier at the latter site. Meristems in temperate and boreal climatic zones have the ability to alternate between active growth and dormancy and the establishment of the dormant state plays a key role in survival under adverse environmental conditions (Olsen et al., 1997; Schrader et al., 2004). In addition to morphological changes of cambial cells during activity-dormancy transition (Druart et al., 2007), other cellular and biochemical changes also take place, such as the accumulation of starch, soluble carbohydrates, lipids and proteins (Sauter and Witt, 1997; Galindo-Gonzalez et al., 2012). Failure to establish the dormant state prior to winter, or precocious activation of growth in the spring, would severely compromise a boreal tree’s ability to survive (Aitken and Adams, 1997; Olsen et al., 1997). Thus, even if environmental conditions at the temperate site LJU in March were favorable for growth, the temperature can still drop below zero, so the trees preventively did not respond to short-term warming.

Repeated sampling of the cambial tissue of *P. sylvestris* and *P. halepensis* from different environments showed that cambial seasonality is a species and site-specific phenomenon. The cambium of *P. sylvestris* from temperate and sub-Mediterranean sites was active during spring and summer but dormant during winter, implying that the cambial seasonal rhythm in this species is homologous at the two sites. In contrast, the cambium of *P. halepensis* exhibited seasonality at the sub-Mediterranean site while regular dormancy was less evident at the Mediterranean site (**Figure 9**). The cambial rhythm of *P. sylvestris* thus does not change when growing in contrasting environmental conditions, whereas in *P. halepensis* cambial rhythm followed weather conditions. The co-existence of different tree species may be reflected in different phenological appearances and different strategies in adapting the rhythm of cambial activity to local environmental conditions (Aljaro et al., 1972; Funada et al., 2016). Waisel and Fahn (1965) already postulated that in well-adapted plants, the annual rhythm of cambial activity usually coincides with the climatic rhythm, which is conservative and may serve as one of the best indicators of the geographical origin of species. According to Creber and Chaloner (1984), the cambial rhythm in trees is genetically controlled but only in the case of favorable conditions. In the case of harsh climatic conditions compared to those of the tree’s native environment, climatic factors may override the genetic ones (Barnett, 1971; Fromm, 2013).

### Cell Differentiation Patterns of *P. halepensis* and *P. sylvestris* in Different Environments

The different cambial rhythms of the two pine species at the site where both species co-exist and typical sites indicate their high but different adaptation strategies. In addition to the cambium, cell differentiation patterns and xylem and phloem formation processes also reflect different rhythms of tree species to function optimally in local environmental conditions (De Luis et al., 2007; Camarero et al., 2010; Oberhuber and Gruber, 2010).

After cell division in the cambium, newly formed xylem and phloem cells undergo differentiation processes, including cell expansion, secondary cell wall formation, and lignification, which are completed with cell death in the case of conducting and supporting cells (Savidge, 2000; Plomion et al., 2001). In addition to the number of cells in the cambium, it is possible to speculate on the cambial state (productive/non-productive) and its cell production rate (high/low productivity) on the basis of the developmental stage of the cells adjacent to the cambium (Barnett, 1971; Prislan et al., 2013). For example, numerous cells in the early stage of development (i.e., cell expansion) next to the cambium indicate its highly productive stage. In contrast, few xylem cells in the stage of secondary wall formation and lignification near the cambium suggest that it stopped producing new cells some time ago. Newly formed xylem cells, namely, continue to develop up to 8 weeks after the cessation of cambial cell production (Gričar et al., 2005).

At the sub-Mediterranean and temperate sites with a clear cambial seasonality, numerous enlarging xylem cells were observed adjacent to the cambium in both species in spring and summer months, demonstrating that the cambium was active. In *P. halepensis* from the Mediterranean site, however, at least one layer of enlarging xylem cells was also observed in the winter months. In addition, we observed latewood tracheids formed in the previous growth season in stages of secondary wall formation and lignification in December 2009 and January 2011 samples. These observations suggest that final processes of differentiation of the last formed cells can continue into the next calendar year (Nix and Villiers, 1985; De Micco et al., 2016). This phenomenon was also noted by Barnett (1971) in *P. radiata*. He reported that terminal latewood tracheids were in late stages of the differentiation process and continued to develop in winter. Based on Barnett’s (1971) observations, it can be inferred that a short pause in cambial productivity of xylem cells does not affect the differentiation processes in these cells. In our Mediterranean samples collected in February, we observed a few enlarging earlywood tracheids, suggesting that the formation of the new xylem increment of 2010 had already started. At that time, the development of the terminal cells in the previous xylem ring was not yet completed, showing that differentiation of the cells of the previous and current xylem increments may overlap.

Periodicity in cambial activity and cell differentiation results in the formation of annual xylem increments with clear earlywood and latewood. In temperate and cold regions, such a wood structure is common due to a unimodal pattern of secondary growth (Camarero et al., 2010; Rathgeber et al., 2011), whereas in Mediterranean climates, secondary growth of conifers genetically does not exhibit a distinct annual cycle with maximum growth in transitional seasons (spring and autumn) and low or no growth in summer. The pattern of xylem ring formation thus follows bimodal rainfall distribution (e.g., De Luis et al., 2007; Camarero et al., 2010; Vieira et al., 2015). Intra-annual density fluctuations (IADFs) or local missing rings regularly occur in xylem as a result of variable climatic conditions for xylem growth (Campelo et al., 2015; Novak et al., 2016b).

According to Gričar et al. (2016), lack of seasonality or absence of true dormancy of the cambium can also be inferred from the structure of the phloem increment. Similar as in xylem, phloem increments in species from temperate and boreal environments consist of early and late phloem, mainly differing in the radial dimension of sieve cells. A tangential band of axial parenchyma cells separates early and late phloem components (Alfieri and Evert, 1968; Gričar and Čufar, 2008). In Mediterranean tree species, however, it is rarely possible to distinguish growth ring boundaries in phloem, since early and late phloem sieve cells do not differ in their radial dimensions and also axial parenchyma cells are often not arranged in tangential bands but are randomly distributed in the phloem (Barnett, 1971; Gričar et al., 2016). We confirmed that the structure of the phloem increments in *P. sylvestris* from the temperate site was typical for such an environment, whereas in *P. sylvestris* from the sub-Mediterranean site, phloem growth ring boundaries were not clearly visible in all cases, mainly due to the scattered distribution of axial phloem parenchyma cells. In *P. halepensis*, phloem growth ring boundaries and the differences between early and late phloem parts were not evident at the (sub)-Mediterranean sites. Lack of growth ring boundaries in the phloem could be ascribed to non-periodical cell differentiation processes on the phloem side. The morphology of xylem and phloem cells is determined by the rate and duration at which developing cambial derivatives expand and form their secondary cell walls (Skene, 1972). The morphology is thus the result of the combined effects of factors that determine developmental patterns and factors that affect the rates of cell division, cell expansion and secondary wall formation (Uggla et al., 2001).

## Conclusion

Our observations revealed the absence of true dormancy in the cambium of *P. halepensis* from Mediterranean areas in Spain. The results thus only partly confirm our hypothesis on the cambial rhythm of the same tree species growing in different environments. In the case of *P. sylvestris*, the cambial rhythm was similar at the typical (temperate) site and at the site where both species co-exist (sub-Mediterranean), while in the case of *P. halepensis*, it differed. In addition to the plasticity of the cambial rhythm, differences in the seasonal dynamics of cell differentiation of xylem and phloem cells, which are reflected in the structure of xylem and phloem increments, is also an important plastic adjustment of trees to environmental heterogeneity. This is crucial for long-term tree performance and survival.

## Author Contributions

JG, KČ, PP, and ML planned and designed the research. PP, KN, and EMC performed the sampling. PP, MŽ, PM, and JŠ contributed during the time consuming sample preparation procedure for observations with light and transmission electron microscope. PP, JG, JŠ, MŽ, US, and GK analyzed the micrographs and interpreted the data. JG and PP wrote the manuscript with contributions from all co-authors.

## Conflict of Interest Statement

The authors declare that the research was conducted in the absence of any commercial or financial relationships that could be construed as a potential conflict of interest.
